# Dr. Rosebrugh’s Ophthalmoscope

**Published:** 1864-07

**Authors:** 


### DR. ROSEBRUGH'S OPHTHALMOSCOPE.

Prof. Helmholtz's immortal discovery of the Ophthalmoscope in 1851, marks the commencement of a new era in Ophthalmic Medicine and Surgery. Up to that time nothing was, or indeed could be known of the appearances of the deep structures of the living eye; now, with this instrument, all parts of the eye, involved in vision, can be brought under the eye of the oculist, and the health or disease of any structure pronounced upon with a certainty in many cases that cannot admit of a doubt; in the case of disease, its particular locality can be perceived and the appropriate treatment, whether medical or surgical, resorted to. In a word, the ophthalmoscope is to the eye what the stethoscope is to the heart and lungs, and the laryngoscope to the throat. It is, however, to be regretted that the time, patience and perseverance necessary in order to use this instrument satisfactorily are such as to prevent its general adoption by the profession; in fact we are of the opinion that up to the present time there are very few expert ophthalmoscopists beyond that limited few who are sufficiently in advance of the text books and the age to be able to keep pace with the advanced ideas of such ophthalmologists as Helmholtz, Von Graefe, Donders and Liebreick.

The new ophthalmoscope of which a full description appeared in this Journal for May, the invention of Dr. Rosebrugh's oculist of Toronto, proposes to do away with the objections to the ordinary ophthalmoscope, to which we have referred.

We have had the privilege of putting Dr. Rosebrugh's instrument to a practical test, and we have great pleasure in reporting that it is all that it claims to be, namely: an ophthalmoscope that will demonstrate the fundus of the eye to any person without any previous knowledge of the mode of using it. In brief, the great advantage of Dr. Rosebrugh's ophthalmoscope consists in *the limited experience necessary in order to use it satisfactorily;* thus placing it within the reach of every medical practitioner.

There is another feature in this new instrument of no little importance, namely: it can be adopted to a small camera obscura upon the ground glass plate of which the image of the fundus oculi can be thrown so as to be seen by a number of persons at the same time; and still farther, a prepared photographic plate being placed in the position of the ground glass plate, photographs can be taken showing the details of the deep structures of the living eye. We have one of these photographs in our possession, showing very clearly the optic nerve enterance and the distribution of the vessels of the retina of a cat.

Dr. Rosebrugh does not claim that his instrument is yet perfect, but that it is based upon the right principle, and is at least a great step in advance; and as the advantages of the ophthalmoscope are now almost universally admitted, we believe the profession will fully appreciate the Doctor's very ingenious invention.

We do with Dr. Rosebrugh most heartily "express the hope that the invention of this instrument will contribute something towards popularizing Ophthalmoscopy, as, in investigating diseases of the eye, the ophthalmoscope is undoubtedly even more essential than the stethoscope in diagnosing diseases of the heart or lungs; and trust its use will aid in banishing from ophthalmic nomenclature the indefinite term of amaurosis, where, as Walther observed, 'the patient sees nothing, and the surgeon likewise—nothing.'"

